# Identification of Survival-Associated miRNA Signatures in Glioblastoma Using Next-Generation Sequencing

**DOI:** 10.3390/ncrna12050041

**Published:** 2026-10-19

**Authors:** Andrea Bakó, Bernadett Torner, Dóra Géczi, András Penyige, Péter Pozsgai, Zsuzsanna Birkó, Álmos Klekner

**Affiliations:** 1 Department of Oncology, Faculty of Medicine, University of Debrecen, H-4032 Debrecen, Hungary; dr.bako.andrea@med.unideb.hu (A.B.); pozsgai.peter@med.unideb.hu (P.P.); 2 Department of Medical Genetics, Faculty of Medicine, University of Debrecen, H-4032 Debrecen, Hungary; torner.bernadett@med.unideb.hu (B.T.); g.dora@med.unideb.hu (D.G.); penyige@med.unideb.hu (A.P.); 3 Department of Neurosurgery, Faculty of Medicine, University of Debrecen, H-4032 Debrecen, Hungary

**Keywords:** GBM-LTS, GBM-STS, miRNA, NGS, brain tissue, biomarker

## Abstract

**Background:** Glioblastoma (GBM) is the most aggressive primary brain tumor in adults and is characterized by pronounced molecular heterogeneity, contributing to poor prognosis and limited therapeutic responsiveness. Distinct clinical subgroups, including short-term survivors (GBM-STS) and long-term survivors (GBM-LTS), differ substantially in disease progression and outcome; however, reliable biomarkers for prognostic stratification remain insufficiently defined. This study aimed to identify microRNA (miRNA) expression patterns capable of distinguishing GBM-STS from GBM-LTS patients. **Methods:** miRNA expression profiling was performed on glioblastoma tissue samples using next-generation sequencing (NGS). Differentially expressed miRNAs between GBM-STS and GBM-LTS groups were subjected to Gene Ontology (GO) Biological Process enrichment analysis to identify dysregulated pathways associated with their predicted target genes. Selected candidate miRNAs were further validated by reverse transcription quantitative polymerase chain reaction (RT-qPCR). **Results:** Functional enrichment analysis revealed significant overrepresentation of pathways related to neurogenesis, neuronal differentiation, and neuron projection development, supporting the involvement of neural developmental programs in glioblastoma progression and invasion. NGS and RT-qPCR analyses demonstrated significant downregulation of hsa-miR-133a-3p, and significant upregulation of hsa-miR-424-5p and has-miR-216a-5p in GBM-STS tissues compared with GBM-LTS and control samples. **Conclusions:** Our findings demonstrate that distinct miRNA expression profiles are associated with survival-related glioblastoma subgroups and may contribute to differences in tumor aggressiveness. Hsa-miR-424-5p, has-miR-216a-5p, and hsamiR-133a-3p may represent promising candidate biomarkers for prognostic stratification in glioblastoma.

## 1. Introduction

Glioblastoma (GBM) is the most aggressive and prevalent primary malignant brain tumor in adults, characterized by rapid proliferation, diffuse infiltration, extensive cellular and molecular heterogeneity, and a poor prognosis despite intensive multimodal therapy [1]. The current standard of care, consisting of maximal safe resection followed by radiotherapy and concomitant temozolomide chemotherapy, yields a median overall survival of approximately 16 months, with less than 10% of patients surviving beyond five years [2,3]. Substantial variability among patients in treatment response and survival highlights the urgent need to identify reliable molecular biomarkers capable of improving prognostic stratification and guiding personalized therapy [4].

Histopathological evaluation, in combination with advanced molecular techniques, has become the current gold standard in glioma diagnostics. Multiple molecular alterations have demonstrated significant diagnostic and prognostic relevance, including mutations in the isocitrate dehydrogenase 1 and 2 (IDH1/2) genes, mutations in the promoter region of telomerase reverse transcriptase (TERT), and methylation of the O6-methylguanine-DNA methyltransferase (MGMT) promoter [5,6]. Despite these molecular classifications, glioblastoma patients with tumors exhibiting comparable histological characteristics and molecular profiles often display substantial variability in overall survival. A more refined stratification of patients could help identify those who would benefit from more intensive treatment and targeted rehabilitation [7].

In recent years, microRNAs (miRNAs)—small non-coding RNAs of approximately 21–23 nucleotides that regulate gene expression at the post-transcriptional level—have emerged as key modulators of oncogenic processes including proliferation, apoptosis, invasion, angiogenesis, and therapeutic resistance [8]. Because of their stability in both fresh-frozen and formalin-fixed paraffin-embedded (FFPE) tissues, as well as in biofluids such as serum and cerebrospinal fluid, miRNAs are promising candidates for use as diagnostic, prognostic, and predictive biomarkers [9]. Previous studies have identified some miRNA signatures associated with GBM patient prognosis. For example, Srinivasan et al. identified a ten-miRNA expression signature capable of stratifying GBM patients into high- and low-risk groups with significantly different survival outcomes using The Cancer Genome Atlas (TCGA) data [10]. Similarly, Hermansen et al. defined a four-miRNA signature (miR-21, miR-219a, miR-196b, and miR-326) that effectively distinguished long-term from short-term survivors, independent of age and MGMT promoter methylation status [7]. However, only a few studies have applied deep sequencing directly to survival-stratified GBM tissue samples.

Previous studies have suggested that ethnicity may represent a potential source of heterogeneity among investigations [11]. Therefore, it is essential to identify significantly deregulated miRNAs that are specific to a patient cohort from a particular geographic region to develop a miRNA panel suitable for the diagnosis of GBM in that specific population.

In the present study, we applied NGS-based small RNA sequencing of GBM tissue specimens obtained from patients who were diagnosed and treated at the Department of Neurosurgery, Faculty of Medicine, University of Debrecen, Hungary, stratified by clinical outcome into short-survival (OS ≤ 17 months) and long-survival (OS > 17 months) groups. We aimed to identify miRNAs differentially expressed between these cohorts, construct predicted miRNA–mRNA regulatory networks and assess their associations with key clinical and molecular parameters. The identification of survival-associated miRNA signatures may contribute to more precise risk assessment and the development of personalized therapeutic strategies in glioblastoma.

## 2. Results

### 2.1. Next-Generation Sequencing (NGS)

To analyze differences in miRNA expression patterns among tissue samples from patients with short-term-survival glioblastoma (GBM-STS), long-term-survival glioblastoma (GBM-LTS), and control samples, next-generation sequencing (NGS) was performed using the Illumina NextSeq 500 platform (Illumina, San Diego, CA, USA). Small RNA-Seq sequencing libraries were generated from total RNA prepared from surgically removed tumor tissue of five GBM-STS, eight GBM-LTS and the peripheral tumor region of four lower-grade (grade 1–2) glioma patients, serving as a control group.

### 2.2. PCA and Clustering Analysis of miRNA Expression in GBM-LTS, GBM-STS and Control Group

**Figure 1:**
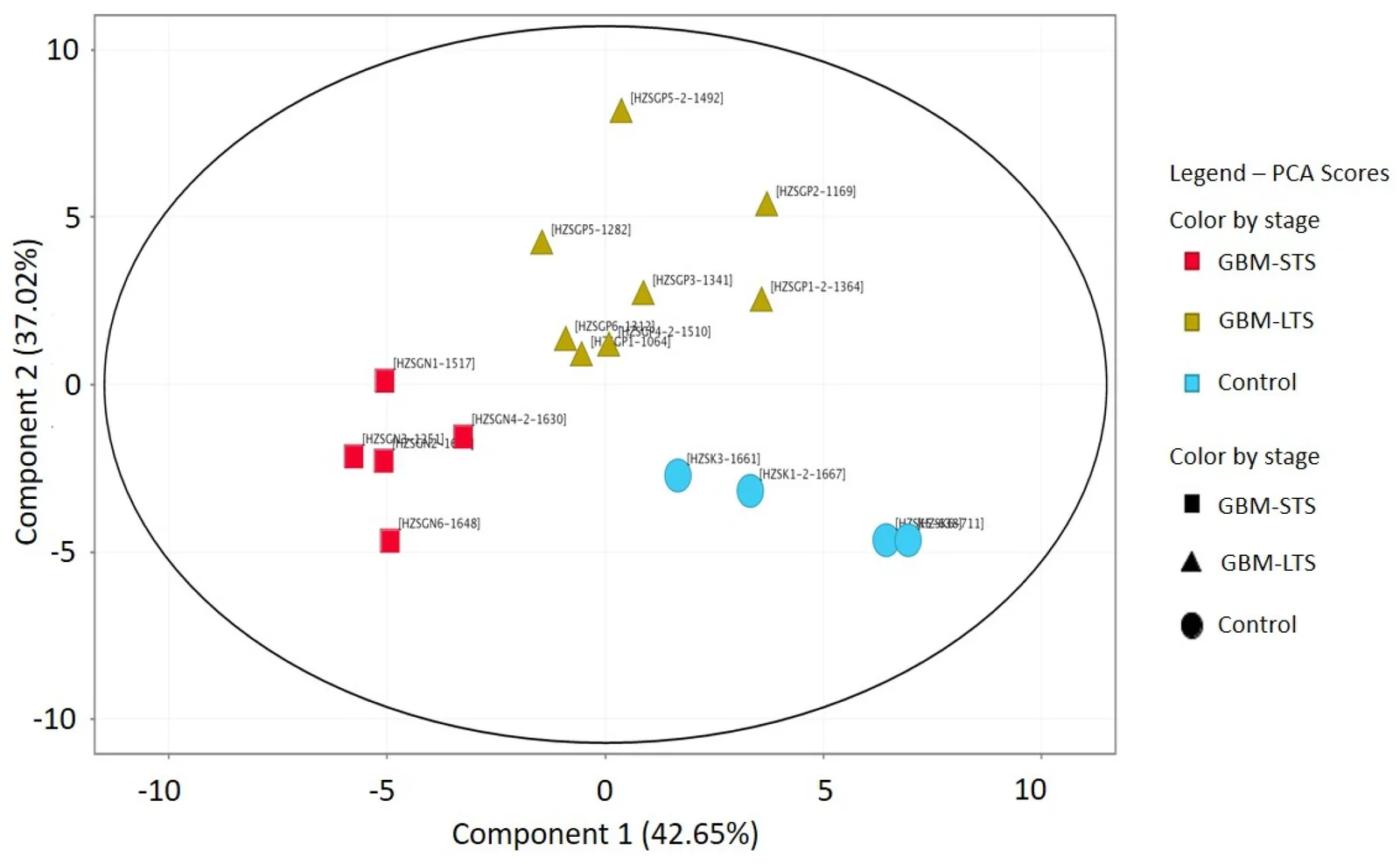
PCA of miRNA expression based on their expression profile. PCA revealed clear separation among the study groups. PC1 (42.65% variance) distinguished GBM-STS (red) and control (blue) samples, while PC2 (37.02%) separated GBM-LTS (yellow) from GBM-STS (red) samples. Samples clustered closely within groups, indicating consistent expression patterns.

To explore the global patterns of miRNA expression and assess the relationships among the study groups, principal component analysis (PCA) and hierarchical clustering were performed. The PCA revealed distinct separation between the analyzed groups (Figure 1). Along the first principal component (PC1), which explains 42.65% of the total variance, the GBM-STS and control samples were clearly distinguished. The second principal component (PC2), accounting for 37.02% of the variance, further separated the GBM-LTS from the GBM-STS samples. Samples belonging to the same group clustered closely together, indicating high intra-group similarity and consistent expression profiles.

One-way ANOVA followed by Tukey’s post hoc test was used to identify miRNAs showing significant differential expression among the study groups based on the significance thresholds of |log_2_ fold change| ≥ 2 and FDR ≤ 0.05. In GBM-STS vs. control samples, 11 miRNAs were downregulated and 10 upregulated (Table S1). The GBM-LTS vs. control comparison revealed 12 downregulated and 6 upregulated miRNAs (Table S2). The GBM-STS vs. GBM-LTS comparison identified 4 downregulated and 12 upregulated miRNAs (Table S3).

**Figure 2:**
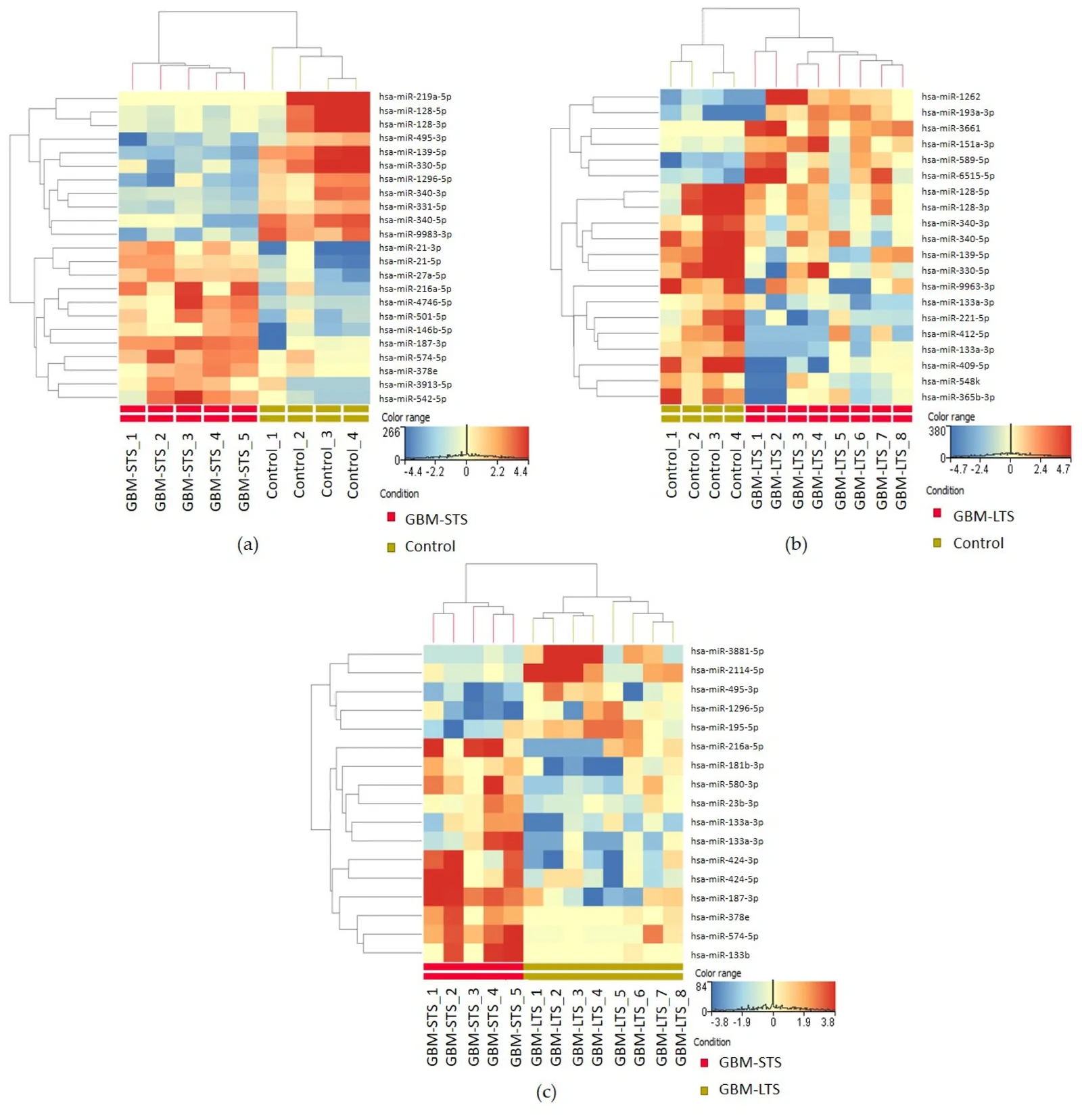
Heatmap analysis of differentially expressed miRNAs across study groups. Heatmaps based on ANOVA illustrate miRNA expression patterns in three pairwise comparisons: (**a**) GBM-STS (red) vs. control (yellow), (**b**) GBM-LTS (red) vs. control (yellow), and (**c**) GBM-LTS (yellow) vs. GBM-STS (red). Distinct expression signatures were observed, with several miRNAs showing significant differences between good- and poor-prognosis groups. Columns represent individual samples (GN—GBM-STS, GP—GBM-LTS, C—control), and rows correspond to individual miRNAs. Upregulated miRNAs are shown in red, and downregulated miRNAs in blue.

The heatmaps in Figure 2 display these significantly altered miRNAs, providing a visual representation of pairwise group differences revealed by the statistical analyses. The heatmap demonstrates clear segregation of the GBM-LTS, GBM-STS, and control samples, reflecting distinct miRNA expression signatures characteristic of each group. GBM-LTS samples clustered together and were well separated from both GBM-STS and control groups, while GBM-STS and control samples formed their own coherent clusters.

Together, the PCA and clustering results consistently highlight substantial differences in miRNA expression patterns between GBM-LTS and GBM-STS samples. These findings suggest that specific miRNA expression signatures are associated with the presence of GBM, reflecting underlying molecular distinctions that correspond to their pathological status.

### 2.3. miRNet-Based Analysis of Deregulated miRNAs

Given that a single miRNA—functioning either as an oncogene or a tumor suppressor—can regulate multiple gene targets, and miRNAs in combination can co-modulate entire signaling pathways, network-based analytical approaches are useful to gain additional information that helps to differentiate between GBM patients with favorable and poor prognoses.

Our analysis relied on experimentally validated miRNA targets using the miRNet platform. We constructed a miRNA-centric network that incorporated direct miRNA–target gene interactions as well as protein–protein interactions (PPIs) among the encoded target proteins.

**Figure 3:**
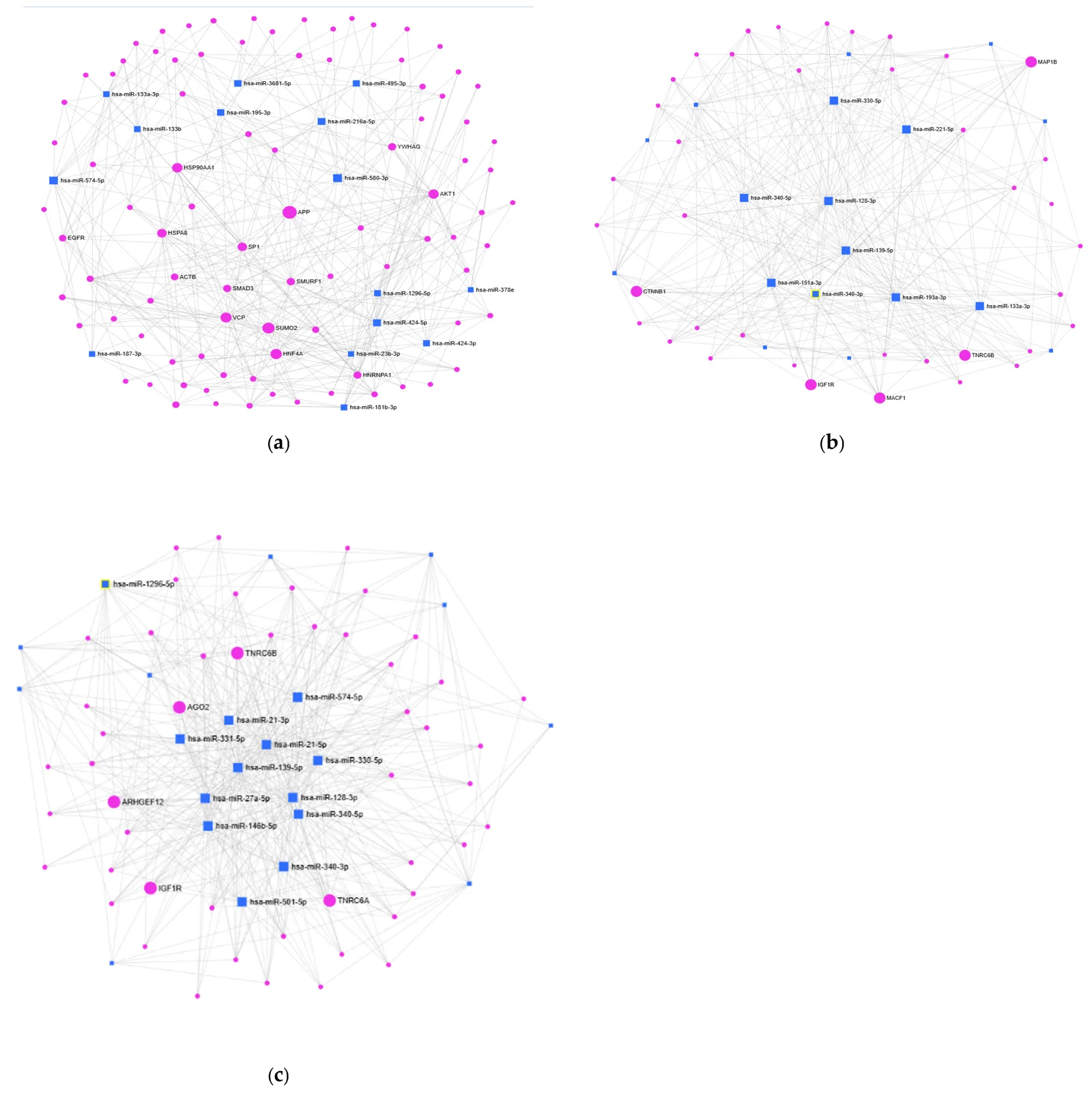
The minimum networks of the deregulated miRNAs and their experimentally validated target genes in GBM-STS–GBM-LTS (**a**), GBM-LTS–control (**b**) and GBM-STS–control (**c**) comparisons. The networks were created by the miRNet tool. Color code: The blue square indicates the differentially expressed miRNAs, while the pink spots represent proteins.

In this heterogeneous network, in the GBM-LTS–GBM-STS comparison hsa-miR-424-5p has the highest degree value (32), followed by hsa-miR-23b-3p (degree value 30) and hsa-miR-216a-5p (degree value 17); in the GBM-LTS–control comparison hsa-miR-128-3p has the highest degree value (degree value 37), followed by hsa-miR-139-5p (degree value 36) and hsa-miR-193a-5p (degree value 27); and in the case of GBM-STS–control hsa-miR-21-5p (degree value 50), hsa-miR-139-5p (degree value 43) and hsa-miR-128-3p (degree value 38) have the most interaction partners, reflecting their importance in the interactome networks. Regarding the target genes, in the GBM-LTS–GBM-STS comparison Amyloid Precursor Protein (APP) was regulated by the highest number of miRNAs and had the most interacting protein partners (degree value 21), followed by protein kinase B α (AKT1) (degree value 20) and Small Ubiquitin-Like Modifier 2 (SUMO2) (degree value 19). In the case of the GBM-LTS–control comparison Microtubule-Associated Protein (MAP1B), trinucleotide repeat-containing adaptor 6B (TNRC6B), and Microtubule Actin Crosslinking Factor 1 (MACF1) (degree value 13) were the highest, while in the case of the GBM-STS–control comparison Argonaute RISC Catalytic Component 2 (AGO2), trinucleotide repeat-containing gene 6A protein (TNRC6A), and IGF1R were the highest. The minimum networks constructed from the miRNA–target gene and protein–protein interactions—created by the miRNet tool—are represented in Figure 3.

### 2.4. Gene Ontology (GO)-Based Analysis of miRNA Targets

**Figure 4:**
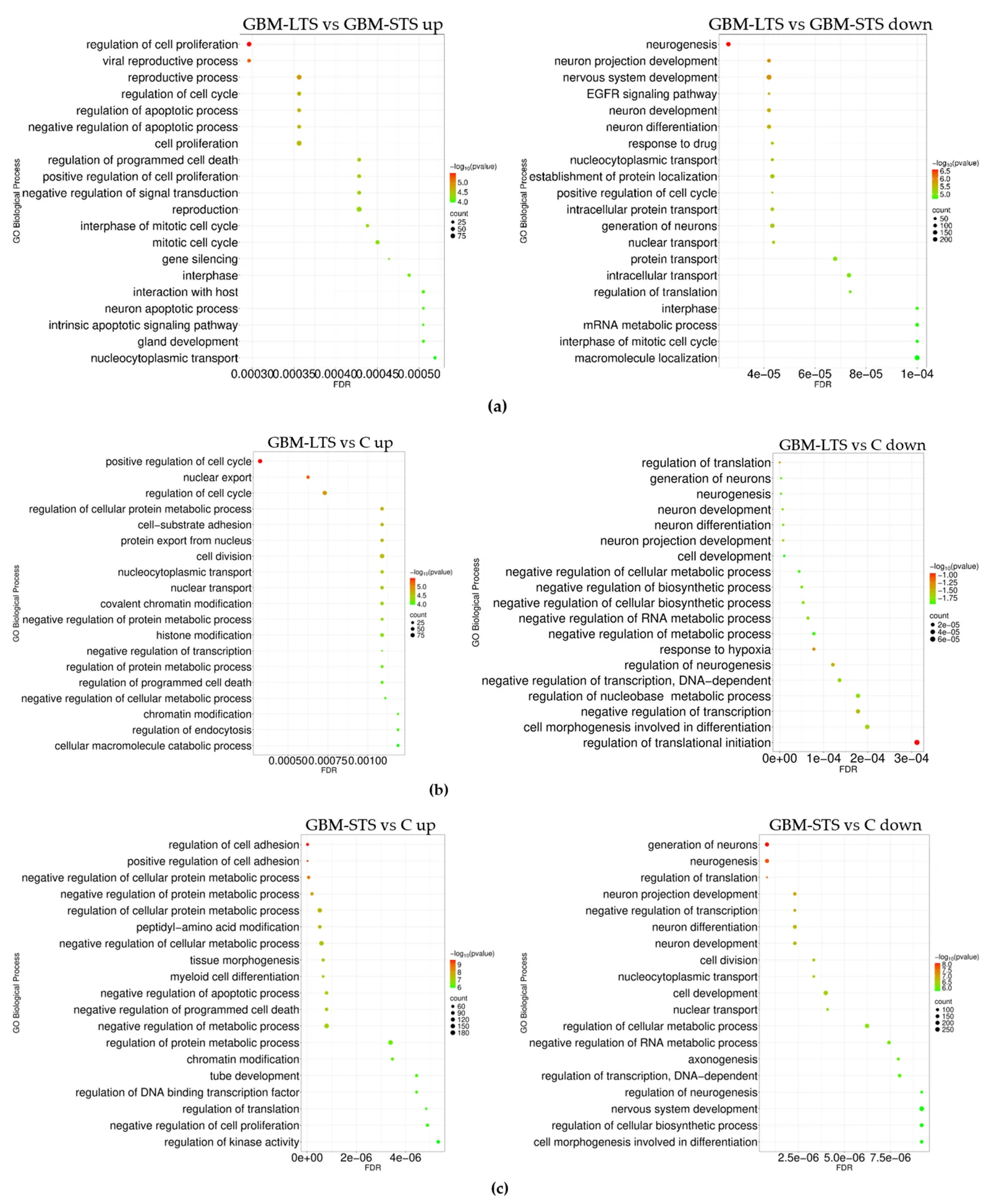
Functional enrichment analysis based on GO Biological Processes was performed for upregulated (figures on the right) and downregulated (figures on the left) miRNA-related target genes in GBM-LTS–GBM-STS (**a**), GBM-LTS–control (**b**) and GBM-STS–control (**c**) comparisons using the NetworkAnalyst 3.0 platform. The significance of the identified biological processes is indicated by their FDR values and −log10 *p*-values, while the dot size reflects the number of genes associated with each process.

Using the GO database in NetworkAnalyst, functional enrichment analysis of the PPI networks derived from experimentally validated miRNA targets revealed several significantly overrepresented biological processes that can be associated with survival outcomes in GBM. In the case of the GBM-LTS–GBM-STS comparison the protein products of the target genes of the upregulated miRNAs were enriched in biological processes such as regulation of cell proliferation, regulation of apoptotic process and signal transduction, while the targets of the downregulated miRNAs are involved in neurogenesis, neuron projection development, neuron differentiation, epidermal growth factor receptor signaling pathway, and drug response (Figure 4a). The targets of upregulated miRNAs in GBM-LTS samples compared to control samples are involved in processes of cell cycle regulation, nuclear export, and regulation of protein metabolic process, while the functions of proteins in association with downregulated miRNAs are related to regulation of translation, neurogenesis, and neuron development (Figure 4b). Regarding the epigenetic regulatory effect of upregulated miRNAs in the GBM-STS–control comparison, the target genes are involved in regulation of cell adhesion and regulation of protein metabolic processes, while the downregulated miRNAs take part in regulation of translation and neurogenesis (Figure 4c).

### 2.5. Clinicopathological Characterization and Kaplan–Meier Survival Analysis of the RT-qPCR Validation Cohort

A total of 40 glioblastoma tissue samples were included in the RT-qPCR validation cohort, comprising 20 GBM-LTS and 20 GBM-STS cases. Clinical parameters of GBM patients are detailed in Table 1. No significant differences between LTS and STS groups were found regarding the laterality, localization, maximal tumor diameter and extension of resection. Furthermore, complex oncotherapy administered to the patients was very similar in both groups, indicating that the different survival times cannot be explained by different oncotherapies. Based on these clinical experiences, identification of further tumor markers would evidently help neuro-oncologists in estimating prognosis.

**Figure 5:**
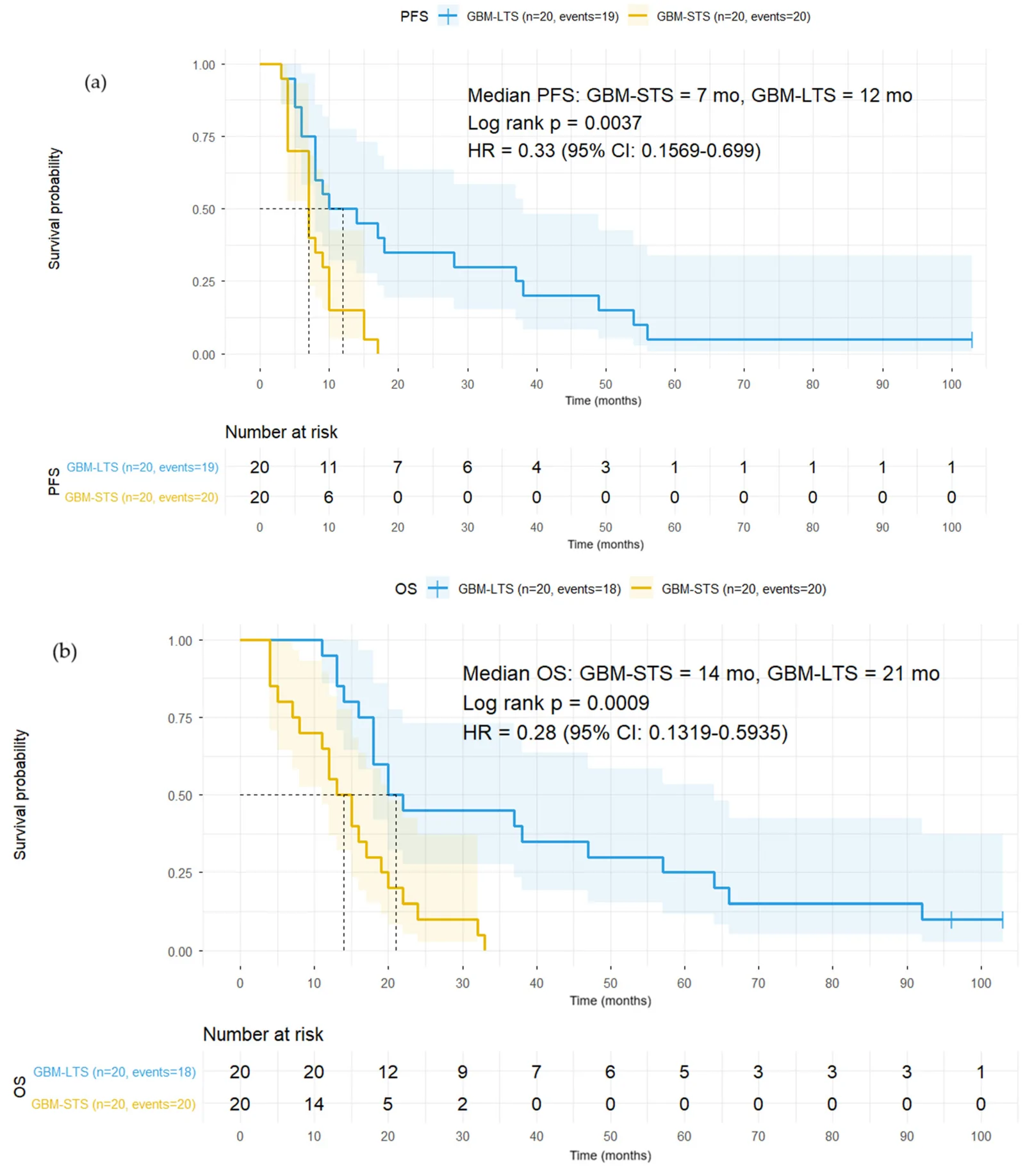
Survival analysis of the RT-qPCR validation cohort in glioblastoma patients. (**a**) Kaplan–Meier overall survival (OS) curves comparing GBM-LTS and GBM-STS patients in the RT-qPCR validation cohort. Median OS was 21 months in the GBM-LTS group and 14 months in the GBM-STS group (log-rank *p* = 0.0009; HR = 0.28, 95% CI: 0.1319–0.5935). (**b**) Kaplan–Meier progression-free survival (PFS) curves comparing GBM-LTS and GBM-STS patients. Median PFS was 12 months in the GBM-LTS group and 7 months in the GBM-STS group (log-rank *p* = 0.0037; HR = 0.33, 95% CI: 0.1569–0.699).

Kaplan–Meier survival analysis demonstrated significantly improved clinical outcomes in the GBM-LTS cohort compared with the GBM-STS cohort. The median overall survival (OS) was 21 months in GBM-LTS patients versus 14 months in GBM-STS patients. Log-rank analysis revealed a statistically significant difference between the two groups (*p* = 0.0009), with a hazard ratio (HR) of 0.28 (95% CI: 0.1319–0.5935), indicating a substantially reduced risk of death in the GBM-LTS cohort (Figure 5a).

Similarly, progression-free survival (PFS) was significantly prolonged in GBM-LTS patients. The median PFS was 12 months in the GBM-LTS group compared with 7 months in the GBM-STS group. Kaplan–Meier analysis showed a significant separation of the survival curves (log-rank *p* = 0.0037), with an HR of 0.33 (95% CI: 0.1569–0.699), demonstrating a lower risk of disease progression or death in the GBM-LTS cohort (Figure 5b).

### 2.6. Validation of Differentially Expressed miR-424-5p, miR-216a-5p and miR-133a-3p by RT-qPCR in Tissue Samples in Combination with Kaplan–Meier Analysis

**Figure 6:**
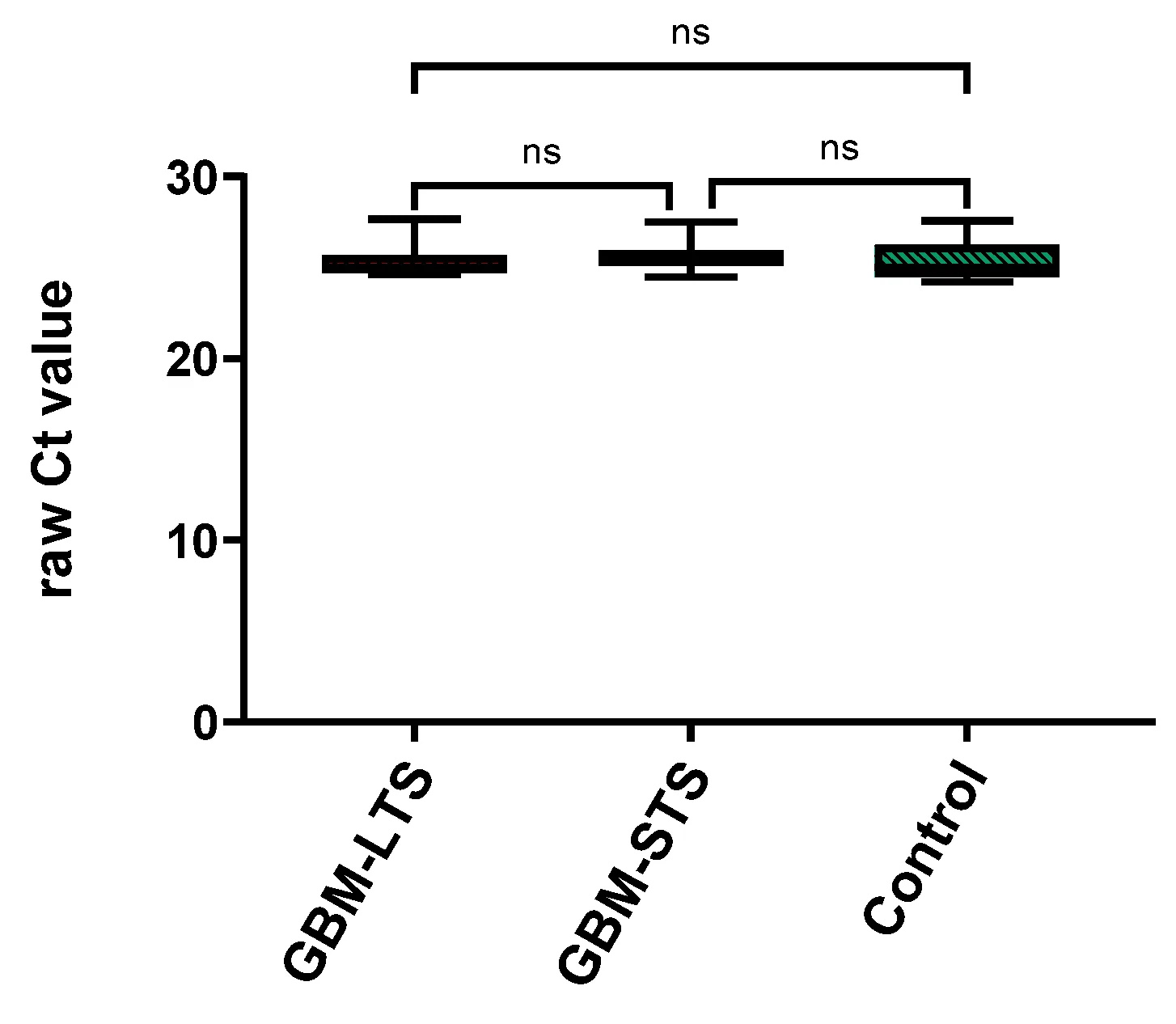
Stability assessment of miR-103a-3p expression across the study groups. Raw Ct values of miR-103a-3p are shown for the three patient groups (n = 20 per group). No significant differences were observed among the groups (Kruskal–Wallis test: H(2) = 3.951, *p* = 0.139). Group medians were 25.00, 25.43, and 24.73, respectively, and Dunn–Bonferroni-adjusted pairwise comparisons were non-significant (ns).

**Figure 7:**
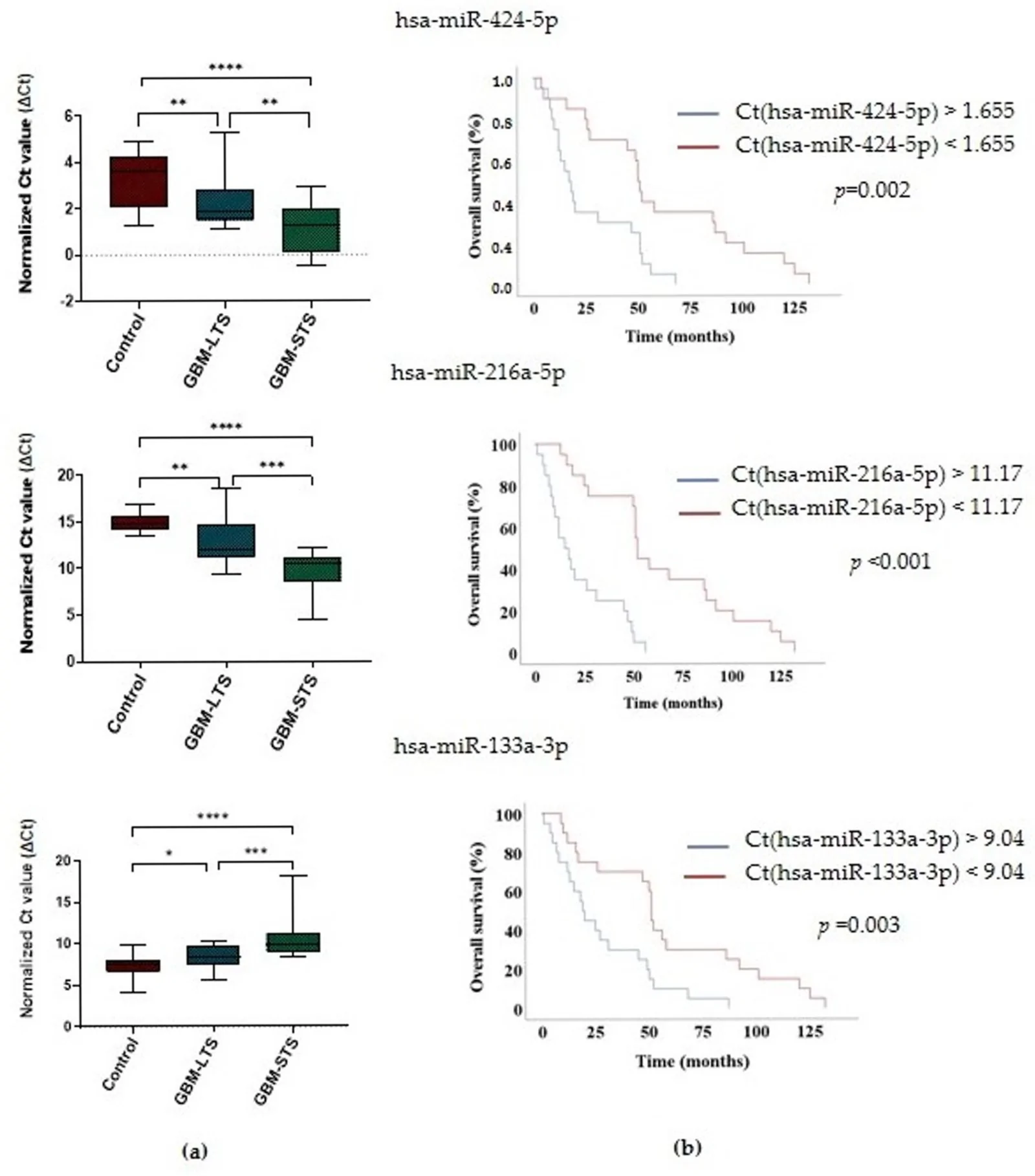
(**a**) RT-qPCR validation of selected differentially expressed miRNAs in GBM-LTS and GBM-STS tissue samples. ΔCt values were calculated as Ct_target miRNA_ − Ct_hsa-miR-103a-3p_; therefore, lower ΔCt values indicate higher miRNA expression, whereas higher ΔCt values indicate lower miRNA expression. Statistical significance between groups was assessed using the Mann–Whitney U test. hsa-miR-424-5p and hsa-miR-216a-5p showed significant upregulation, while hsa-miR-133a-3p showed significant downregulation in GBM-LTS samples compared with GBM-STS and control samples. *p* < 0.05 (*), *p* < 0.01 (**), *p* < 0.001 (***), *p* < 0.0001 (****). (**b**) Overall survival (OS) of GBM patients with low or high hsa-miR-424-5p, hsa-miR-216a-5p and hsa-miR-133a-3p expression levels.

To approve the results obtained by NGS (using the predefined thresholds of |log_2_ fold change| ≥ 2 and FDR ≤ 0.05) on tissue samples of GBM-LTS and GBM-STS in a larger cohort, three differentially expressed miRNAs were selected for RT-qPCR validation. These included the upregulated hsa-miR-424-5p (logFC = 2.52) and hsa-miR-216a-5p (logFC = 2.57), as well as the downregulated hsa-miR-133a-3p (logFC = −1.94), identified in the GBM-STS vs. GBM-LTS comparison. miRNA expression levels were normalized to hsa-miR-103a-3p. The raw Ct values of hsa-miR-103a-3p did not differ significantly among the control, GBM-LTS, and GBM-STS groups (Kruskal–Wallis test: H(2) = 3.951, *p* = 0.139; Figure 6). The Ct distributions displayed substantial overlap, and all Dunn–Bonferroni-adjusted pairwise comparisons were non-significant. These findings demonstrate the stable expression of hsa-miR-103a-3p across the study groups and support its suitability as an endogenous normalization control. All assays were performed in technical triplicates. Statistical analysis using the Mann–Whitney U test confirmed significant upregulation of hsa-miR-424-5p and hsa-miR-216a-5p, together with significant downregulation of hsa-miR-133a-3p in GBM-LTS samples compared with both GBM-STS and control samples (Figure 7a). Additionally, we performed Kaplan–Meier analysis, which proved that GBM patients with lower hsa-miR-424-5p and hsa-miR-216a-5p expression levels had significantly shorter OS (*p* = 0.002 and *p* < 0.001 respectively), while the decreased hsa-miR-133a-3p expression level showed association with significantly shorter OS (*p* = 0.003) (Figure 7b).

**Figure 8:**
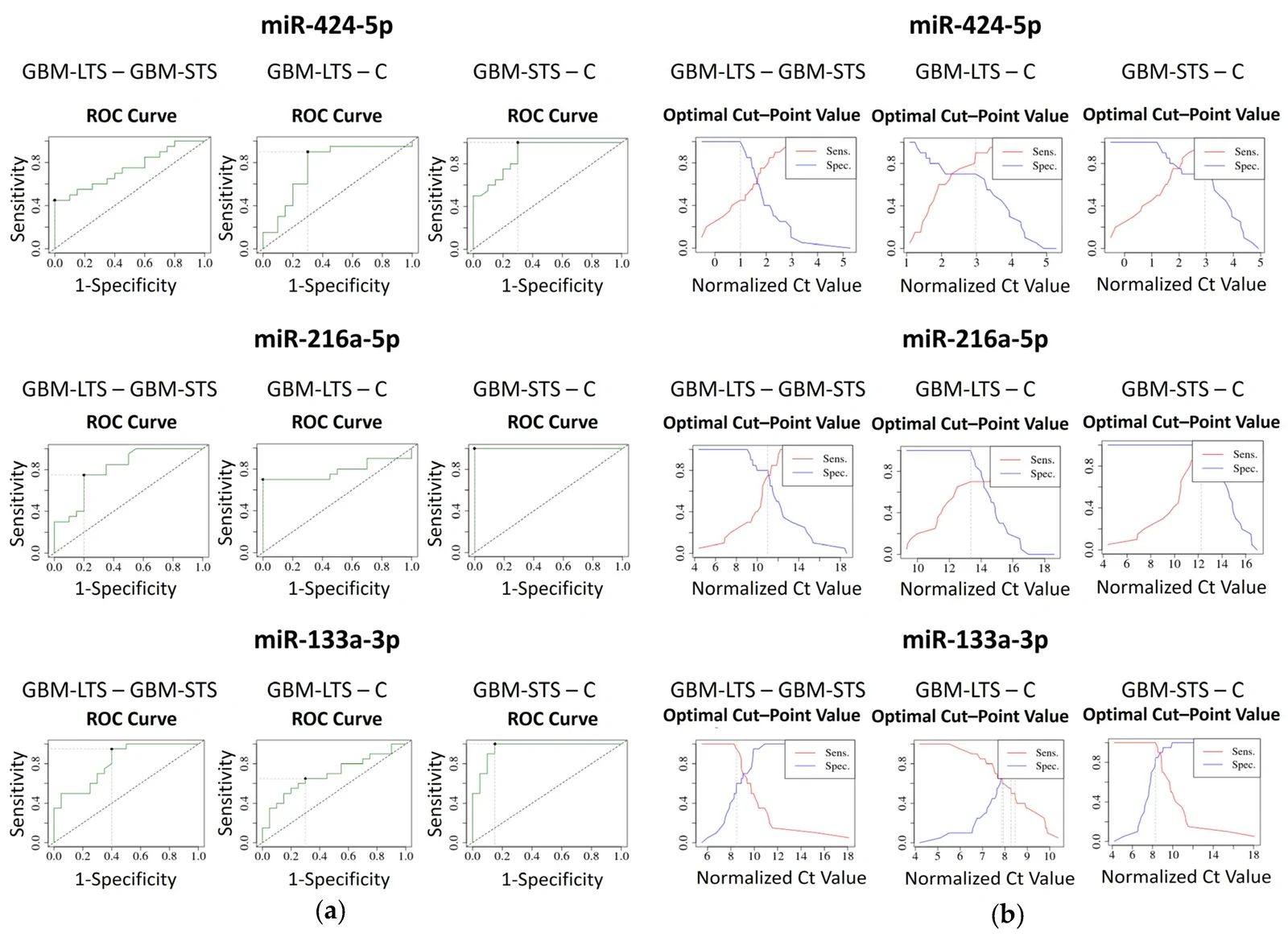
(**a**) ROC (receiver operating characteristic) curves with AUC (area under the curve) were prepared to analyze and represent the sensitivity and specificity of hsa-miR-424-5p, hsa-miR-216a-5p and hsa-miR-133a-3p in GBM-LTS–GBM-STS, GBM-LTS–control and GBM-STS–control comparisons. (**b**) Calculated optimal cut-point values of hsa-miR-424-5p, hsa-miR-216a-5p and hsa-miR-133a-3p in GBM-LTS–GBM-STS, GBM-LTS–control and GBM-STS–control comparisons.

Subsequently, receiver operating characteristic (ROC) analysis was performed to evaluate the diagnostic value of hsa-miR-424-5p, hsa-miR-216a-5p, and hsa-miR-133a-3p (Figure 8). For this analysis, normalized Ct values obtained from RT-qPCR were compared among the GBM-LTS, GBM-STS, and control groups. The calculated area under the curve (AUC), specificity, sensitivity and optimal cut-off values for all three miRNAs in the comparisons of GBM-LTS and GBM-STS, GBM-LTS and control, and GBM-STS and control are presented in Table 2. These results indicate that hsa-miR-424-5p, hsa-miR-216a-5p, and hsa-miR-133a-3p may serve as potential biomarkers for distinguishing GBM-STS from GBM-LTS.

## 3. Discussion

Glioblastoma (GBM) is the most aggressive primary brain tumor in adults, characterized by pronounced molecular and cellular heterogeneity, which contributes to its poor prognosis and limited therapeutic responsiveness [12,13]. Recent studies have further refined GBM classification by distinguishing biologically and clinically relevant subtypes, including short-term survivors (GBM-STS) and long-term survivors (GBM-LTS), which differ markedly in disease progression and treatment outcomes [14,15]. Despite these advances, reliable biomarkers capable of stratifying patients into these prognostic groups remain insufficiently defined.

MicroRNAs (miRNAs) have emerged as key post-transcriptional regulators involved in tumor development, progression, and therapy response, making them promising candidates for biomarker discovery in GBM [16,17]. Therefore, the aim of the present study was to identify miRNA expression patterns in tumor tissue samples that can effectively discriminate between GBM-STS and GBM-LTS patient groups. Such molecular markers could contribute to improved prognostic stratification and support more personalized therapeutic decision-making.

### 3.1. Biological Process Enrichment Analysis of miRNA Targets

The Gene Ontology (GO) Biological Process enrichment analysis of targets of differentially expressed miRNAs between GBM-LTS and GBM-STS groups on the base of the results of miRNA NGS revealed a significant overrepresentation of pathways associated with neurogenesis, neuron projection development, and neuronal differentiation and development. Neurogenesis-related pathways have increasingly been implicated in glioblastoma progression, particularly in tumors arising near neurogenic niches such as the subventricular zone. These regions contain neural stem and progenitor cells, and their involvement has been linked to more aggressive tumor behavior and poorer clinical outcomes [18].

In line with the regulatory role of miRNAs in neurogenesis-associated pathways, our NGS analysis demonstrated a more pronounced downregulation of hsa-miR-128 expression in the GBM-STS group compared with the GBM-LTS group. Reduced hsa-miR-128 expression has been linked to the upregulation of BMI1 and other Polycomb Repressive Complex (PRC)-associated factors, including SUZ12, which promote glioblastoma proliferation, stemness maintenance, therapy resistance, and self-renewal of neural stem cells (NSCs) and glioma stem cells (GSCs) [19,20]. Neuron projection development, including axonogenesis and dendritic growth, may further contribute to tumor progression by enhancing cell migration and invasion [21]. Glioblastoma cells can hijack neuronal developmental programs to infiltrate the surrounding brain parenchyma, thereby limiting complete surgical resection and contributing to recurrence [22,23]. Several dysregulated miRNAs are involved in these invasion-associated processes. For example, hsa-miR-21 promotes invasiveness through suppression of Reversion-Inducing Cysteine-Rich Protein With Kazal Motifs (RECK) and tissue inhibitor of metalloproteinases 3 (TIMP3), leading to matrix metalloproteinase (MMP) activation and extracellular matrix degradation [24]. Similarly, downregulation of hsa-miR-133a increases MMP9 expression and facilitates glioma invasion and migration [25]. In our study, hsa-miR-133a showed significant differential expression between the GBM-STS and GBM-LTS groups in both NGS profiling and RT-qPCR validation, supporting its potential role in glioblastoma aggressiveness and disease stratification.

Additionally, pathway enrichment analysis of the predicted targets of upregulated miRNAs in GBM-STS patients revealed a significant overrepresentation of the “negative regulation of cellular protein metabolic process” pathway. Since miRNAs primarily function through translational repression and mRNA destabilization, enrichment of pathways associated with negative regulation of protein metabolic processes likely reflects a broader proteostatic reprogramming that supports glioblastoma progression and therapy resistance [26]. For example, hsa-miR-424 as a member of our validation group has been linked to metabolic reprogramming through modulation of hypoxia-inducible factor 1 alpha (HIF-1α) stability and tricarboxylic acid cycle-associated enzymes, supporting adaptation to hypoxic tumor conditions [27]. Furthermore, the members of the miR-216 family (hsa-miR-216a-5p is the member of the validation group) have been associated with regulation of autophagy, phosphoinositide 3-kinase (PI3K)/AKT signaling, and transforming growth factor beta (TGF-β)-mediated cellular responses in different cancer models [28].

### 3.2. Validation of Differentially Expressed miR-424-5p, miR-216a-5p and miR-133a-3p by RT-qPCR

The altered expression patterns of hsa-miR-424-5p, hsa-miR-216a-5p, and hsa-miR-133a-3p among the control, GBM-STS and GBM-LTS groups were further validated by RT-qPCR analysis. On the base of that, miR-424-5p and miR-216a-5p displayed significantly increased expression in GBM-STS samples compared with both GBM-LTS and control tissues.

In the present study, as the results of the validation process, both miR-424-5p and miR-216a-5p showed significantly increased expression in GBM-STS tissue samples compared to both GBM-LTS and control tissues. These findings appear to contrast with several previous studies that predominantly described tumor-suppressive roles for these miRNAs in glioblastoma. In particular, Jin et al. demonstrated in 93 glioma tumor tissues and 55 matched adjacent non-neoplastic brain tissues that miR-424 expression was significantly reduced in glioma and was associated with higher tumor grade and poorer Karnofsky Performance Status [29]. Zhao et al. similarly reported reduced miR-424 expression in glioma tissues, with lower levels associated with poorer overall survival [30]. Furthermore, miR-424-5p has been reported to inhibit glioma progression through suppression of bifunctional apoptosis regulator (BFAR), AKT Serine/Threonine Kinase 1 (AKT1) and RAF1-mediated signaling [31]. Previous studies have demonstrated that miR-216a-5p exerts tumor-suppressive effects in glioma by inhibiting cell proliferation, migration, and invasion via targeting of leucine-rich repeat-containing G protein-coupled receptor 5 (LGR5) and modulation of the miR-216a/AQP4 regulatory axis [32,33]. Similarly, Peng et al. demonstrated reduced miR-216a expression in glioma tissues and identified a LINC00461/miR-216a/AQP4 regulatory axis associated with malignant glioma phenotypes. Inhibition of this pathway reduced glioma cell proliferation, migration, invasion, and chemoresistance, with supportive findings in a xenograft model [34].

In contrast, oncogenic effects of miR-424-5p and miR-216a-5p have been reported in other type of malignancies. Ding et al. demonstrated that miR-424-5p promoted proliferation, migration, and survival of hepatocellular carcinoma through activation of the PI3K/AKT signaling pathway [35]. Similarly, Xu et al. reported significantly elevated miR-424 expression in melanoma tissues and cell lines, where increased expression correlated with advanced clinical stage, lymph node metastasis, and poor prognosis [36]. Furthermore, in gastric cancer, miR-216a-5p promoted tumor progression and metastatic potential through regulation of the miR-216a-5p/FDFT1 axis [37].

Accumulating evidence suggests that the biological functions of miRNAs are highly context-dependent and may vary according to the molecular background, signaling landscape, and tumor microenvironment [38,39]. Furthermore, glioblastoma is characterized by substantial intra- and intertumoral heterogeneity, which may profoundly alter miRNA-mediated regulatory networks and contribute to divergent functional consequences across patient cohorts [39]. An additional explanation for the discrepancy between our findings and previous reports may involve population-specific molecular differences. Notably, several studies reporting tumor-suppressive functions and reduced miR-424 expression in glioblastoma were conducted predominantly in Asian patient cohorts [29,30,31,32,33,34]. Emerging evidence indicates that miRNA expression patterns may differ across ethnic populations, potentially influencing downstream regulatory interactions and the biological consequences of miRNA dysregulation [40,41]. Therefore, ethnicity-associated molecular heterogeneity may partly contribute to the distinct miRNA expression profiles observed in our cohort.

In contrast to miR-424-5p and miR-216a-5p, the expression pattern of miR-133a-3p observed in our cohort was consistent with previous literature data. We detected significant downregulation of miR-133a-3p in GBM-STS samples compared to both GBM-LTS and control tissues, supporting its proposed tumor-suppressive role in glioblastoma. Sakr et al. reported that miR-133a suppresses glioma progression by directly targeting membrane-type 1 matrix metalloproteinase (MT1-MMP), thereby reducing invasive capacity [42]. DNA methylation-mediated loss of miR-133a expression correlated with accelerated glioma progression and poorer prognosis, while restoration of expression inhibited cell proliferation and colony formation in vitro [43]. However, one earlier report suggested that miR-133a may also modulate apoptosis resistance mechanisms in GBM through suppression of DR5 and activation of nuclear factor kappa B (NF-κB) signaling [44].

To further investigate whether the differential expression observed between GBM-LTS and GBM-STS was reproducible in an independent dataset, we performed a comparative analysis of miR-424-5p, miR-216a-5p, and miR-133a-3p expression using publicly available TCGA-GBM data. Patients were stratified into GBM short-term survivors (GBM-STS) and GBM long-term survivors (GBM-LTS) using a 17-month overall survival cut-off. On the base of the analysis, none of the three miRNAs showed a statistically significant difference between GBM-LTS and GBM-STS in the TCGA dataset. Thus, the TCGA analysis did not independently reproduce the differential expression observed in our cohort, suggesting that the association of these miRNAs with long- versus short-term survival may be cohort- or context-dependent. Importantly, this finding does not contradict previous reports describing differential miRNA expression between GBM and non-neoplastic brain tissue, as these analyses address a different biological comparison.

In this context, Grigore et al. recently performed an integrative analysis of publicly available miRNA expression datasets comprising 743 adult GBM samples and 59 non-cancerous brain samples and identified 209 significantly deregulated miRNAs, including 115 upregulated and 94 downregulated miRNAs. Notably, miR-424-5p was among the miRNAs upregulated in GBM compared with non-cancerous brain tissue [45]. This independent observation is consistent with the increased miR-424-5p expression observed in our cohort and provides additional evidence that miR-424-5p is associated with GBM biology. However, because the Grigore et al. study compared GBM with non-neoplastic brain tissue rather than GBM-LTS with GBM-STS, its findings cannot be directly interpreted as evidence for an association between miR-424-5p expression and survival duration. Together with our TCGA analysis, these data suggest that miR-424-5p dysregulation may be associated with the GBM phenotype, whereas its relationship with long- versus short-term survival may depend on the characteristics of the studied cohort.

Collectively, the three miRNAs analyzed in this study illustrate the dual and context-dependent roles of miRNAs in glioblastoma. miR-216a-5p and miR-424-5p may act oncogenically in aggressive tumors while showing tumor-suppressive activity in other contexts, whereas miR-133a-3p predominantly functions as a tumor suppressor. Such dual functionality is likely mediated by differences in target gene networks, tumor molecular subtypes, and disease stage. These observations underscore the importance of patient- and tumor-specific miRNA profiling for understanding glioblastoma biology and for evaluating their potential as prognostic biomarkers or therapeutic targets.

## 4. Materials and Methods

### 4.1. Patients and Samples

Based on the 16.6-month median overall survival time of all glioblastoma patients treated at the Department of Neurosurgery, Faculty of Medicine, University of Debrecen (Hungary), patients were divided into two groups. Patients with overall survival (OS) more than 17 months formed the long-term survival group (GBM-LTS), while patients with OS shorter than or equal to 17 months composed the short-term survival group (GBM-STS). The median overall survival time for the GBM-LTS and GBM-STS groups was 32.5 months (95% CI: 22–64) and 12.0 months (95% CI: 11–15), respectively. Inclusion criteria for all patients were pathohistologically proven IDH wild-type glioblastoma, postoperative Karnofsky performance scale at least 70 and completed concurrent chemo-irradiation therapy after tumor removal (6 weeks of focal brain radiotherapy, 60 Gy delivered in 2 Gy fractions with concurrent oral administration of 75 mg/m^2^/day temozolomide).

Control specimens were obtained from patients with lower-grade glioma (grade 1–2). Aiming at radical removal of the tumor, the peritumoral area was also resected according to the general guidelines of neurosurgical procedures. These tumor-adjacent tissue samples were collected intraoperatively, and histopathological evaluation confirmed the absence of tumor cells in the sampled peripheral tissue. All samples were histologically verified, snap-frozen immediately after resection, and stored at −80 °C until analysis.

All participants provided written informed consent, and clinical data were retrieved from medical records. No patient received chemotherapy or radiotherapy prior to sample collection.

For miRNA next-generation sequencing (NGS), patients in the GBM-STS and GBM-LTS groups were aged 50–67 years (mean: 59 and 62 years, respectively), while controls ranged from 52 to 72 years (mean: 64 years) (Table 3). In the validation cohort, controls (n = 20) were aged 40–76 years (median: 66 years), GBM-STS patients (n = 20) 33–81 years (median: 59 years), and GBM-LTS patients (n = 20) 37–75 years (median: 57 years). Sex distribution was balanced across all groups.

The study was approved by the Scientific and Research Ethics Committee of the Medical Research Council (ETT TUKEB; IV/1753-/2021/EKU) and conducted in accordance with the Declaration of Helsinki.

### 4.2. Tissue Processing and RNA Isolation for Next-Generation Sequencing (NGS)

Tissue specimens were kept on ice during dissection and subsequently homogenized using a MagNa Lyser system (Roche Ltd., Basel, Switzerland). Total RNA, encompassing small RNA fractions, was extracted with the miRNeasy Mini Kit (Qiagen, Hilden, Germany) following the manufacturer’s protocol. A total of 30 mg of tissue was processed per sample. RNA concentration and purity were evaluated using a NanoDrop spectrophotometer (Thermo Scientific, Waltham, MA, USA).

### 4.3. miRNA Sequencing and Bioinformatics Analysis

To generate a comprehensive miRNA expression profile, small RNA sequencing was carried out using the Illumina NextSeq 500 platform. All experimental steps, including library construction, sequencing, and initial data processing, were performed at the Genomic Medicine and Bioinformatics Core Facility (Department of Biochemistry and Molecular Biology, Faculty of Medicine, University of Debrecen). Five GBM-STS, eight GBM-LTS and four control patient biological replicates were included in the analysis.

RNA quality was assessed using an Agilent Bioanalyzer equipped with the Eukaryotic Total RNA Nano Kit, in accordance with the manufacturer’s guidelines. Only samples with an RNA integrity number (RIN) greater than 7 were selected for library preparation. Small RNA libraries were generated from 1 µg of total RNA using the NEBNext Multiplex Small RNA Library Prep Set for Illumina (1–48) 96 rxn kit (New England BioLabs, Ipswich, MA, USA) following the supplier’s protocol. Library size distribution and concentration were verified using the Agilent Bioanalyzer DNA 1000 chip (Agilent Technologies, Santa Clara, CA, USA).

Sequencing was performed in single-end mode with a read length of 50 bp on the Illumina NextSeq 500 system (Illumina, San Diego, CA, USA). Raw sequencing reads (FASTQ format) were aligned to the human reference genome (GRCh38) using the Novoalign aligner, and alignment files were generated in BAM format. Subsequent data processing and normalization were conducted using StrandNGS software (www.strand-ngs.com version 2.8, build 230243; Strand Life Sciences, Bangalore, India), applying the DESeq normalization method, to correct for differences in sequencing depth and library size among samples. The DESeq algorithm was used exclusively as a normalization procedure, whereas differential expression testing was subsequently performed on the normalized expression values within the StrandNGS workflow.

Differential expression analysis was performed using one-way analysis of variance (ANOVA) to assess overall differences in miRNA expression between groups [46]. Prior to analysis, normalized expression values were log_2_-transformed to approximate normal distribution. Homogeneity of variances was evaluated, and where applicable, assumptions of ANOVA were considered satisfied. This approach enabled the statistical comparison of normalized expression profiles across the predefined study groups and subsequent pairwise evaluation of group-specific differences.

Following a significant overall ANOVA result, Tukey’s honestly significant difference (HSD) post hoc test was applied for multiple pairwise comparisons [47]. To control for multiple testing, *p*-values were adjusted using the Benjamini–Hochberg procedure, and the false discovery rate (FDR) was calculated [48]. miRNAs were considered significantly differentially expressed if they met the criteria of |log_2_ fold change| ≥ 2 and FDR ≤ 0.05.

### 4.4. miRNA Target Prediction, Network Construction, and Functional Enrichment Analysis

Experimentally validated target genes of differentially expressed (DE) miRNAs were identified using the web-based platform miRNet (http://www.mirnet.ca; accessed on 12 December 2022) based on the miRTargetLink (https://ccb-compute.cs.uni-saarland.de/mirtargetlink2/, accessed on 12 December 2022) database [49] for miRNA–target interaction information. The miRNA–target interaction network was generated using miRNet, and key miRNAs were prioritized based on degree centrality measures.

Protein–protein interaction (PPI) networks were established using the 50 most significantly upregulated and downregulated mRNAs with the NetworkAnalyst 3.0 [50] platform (https://www.networkanalyst.ca). Hub proteins within the PPI networks were identified and ranked according to their degree centrality scores.

Network-based Gene Ontology (GO) functional annotation and pathway enrichment analyses were performed using NetworkAnalyst 3.0, employing the Kyoto Encyclopedia of Genes and Genomes (KEGG) database for pathway enrichment. Statistical significance was defined as a *p*-value < 0.05.

### 4.5. RT-qPCR-Based Validation of Selected miRNAs Identified by miRNA-Seq

For validation, total RNA was isolated from 30 mg tissue specimens obtained from 20 controls, 20 GBM-LTS and 20 GBM-STS patients using the miRNeasy Mini Kit (Qiagen, Hilden, Germany), following the manufacturer’s instructions. RNA concentration and purity were assessed with a NanoDrop spectrophotometer (Thermo Scientific, Waltham, MA, USA).

Mature miRNA levels were quantified using the miRCURY LNA miRNA PCR Assay (Qiagen). Reverse transcription was performed with the miRCURY LNA RT Kit (Qiagen) under the following conditions: incubation at 42 °C for 60 min, followed by enzyme inactivation at 95 °C for 5 min.

Quantitative real-time PCR (RT-qPCR) was carried out on a LightCycler^®^ 96 system (Roche Ltd., Pleasanton, CA, USA) using the miRCURY LNA SYBR Green PCR Kit (Qiagen). The expression levels of hsa-miR-424-5p, hsa-miR-216a-5p and hsa-miR-133a-3p were determined in GBM-LTS, GBM-STS and control samples.

Thermal cycling conditions consisted of an initial activation step at 95 °C for 120 s, followed by 45 amplification cycles, each including denaturation at 95 °C for 10 s and combined annealing/extension at 56 °C for 60 s. Melting curve analysis was subsequently performed with fluorescence acquisition at 95 °C for 20 s, 40 °C for 20 s, and 85 °C for 1 s. The reaction concluded with a cooling step at 37 °C for 30 s.

miRNA expression levels were calculated using the comparative cycle threshold (Ct) method. hsa-miR-103a-3p served as the endogenous control. ΔCt values were calculated by subtracting the mean Ct value of hsa-miR-103a-3p from the Ct value of each target miRNA. These ΔCt values were used for statistical analysis. Accordingly, lower ΔCt values indicate higher miRNA expression, whereas higher ΔCt values indicate lower miRNA expression.

### 4.6. Statistical Analysis

The stability of hsa-miR-103a-3p was assessed by comparing raw Ct values among the control, GBM-LTS, and GBM-STS groups using the Kruskal–Wallis test followed by Dunn–Bonferroni pairwise analysis.

Statistical evaluation of RT-qPCR-derived miRNA data was performed following assessment of data distribution using the Kolmogorov–Smirnov test. Differences in expression levels between groups were analyzed using the nonparametric Mann–Whitney U test in GraphPad Prism (version 8.0.1). A *p*-value < 0.05 was considered statistically significant.

Receiver operating characteristic (ROC) curves and corresponding area under the curve (AUC) values were generated using easyROC software (version 1.3.1; http://biosoft.erciyes.edu.tr/app/easyROC/; accessed on 25 July 2016) [51]. ROC analysis was conducted to determine optimal cut-off values by identifying the best balance between sensitivity and specificity.

Survival analysis was performed using the Kaplan–Meier method [52], and differences between groups were evaluated using the log-rank test.

Statistical significance was set at *p* < 0.05 for all analyses.

## 5. Conclusions

In summary, our study identified distinct miRNA expression patterns associated with survival differences in glioblastoma patients and demonstrated that specific miRNAs may contribute to the biological divergence between GBM-STS and GBM-LTS groups. Functional enrichment analysis of differentially expressed miRNA targets revealed significant involvement of neurogenesis-associated pathways, neuronal differentiation, and neuron projection development, supporting the concept that glioblastoma exploits neural developmental programs to promote tumor progression, invasion, and recurrence.

Among the investigated miRNAs, hsa-miR-133a-3p showed consistent downregulation in GBM-STS samples in both NGS profiling and RT-qPCR validation, supporting its proposed tumor-suppressive role in glioblastoma. In contrast, hsa-miR-424-5p and hsa-miR-216a-5p displayed significantly increased expression in GBM-STS tissues, suggesting potential oncogenic functions in aggressive glioblastoma subgroups. These findings differ from several previous reports describing tumor-suppressive effects of these miRNAs in GBM, highlighting the context-dependent nature of miRNA-mediated regulation and the substantial molecular heterogeneity of glioblastoma. Differences in tumor molecular background, signaling networks, tumor microenvironment, and potentially ethnicity-associated molecular variability may all contribute to these divergent observations.

Collectively, our results emphasize the complex and dual roles of miRNAs in glioblastoma biology and support the potential utility of miRNA expression profiling for prognostic stratification. In particular, miR-424-5p, miR-216a-5p, and miR-133a-3p may represent promising candidate biomarkers for distinguishing aggressive short-term-survivor tumors from more favorable long-term-survivor cases. Nevertheless, further large-scale and mechanistic studies are required to validate their biological significance and to clarify their potential applicability as prognostic biomarkers or therapeutic targets in glioblastoma.

## Figures and Tables

**Table 1:** Characteristics of GBM patients according to laterality, localization, maximal diameter of tumor, extension of resection and therapy.

	GBM-STS (n = 20)	GBM-LTS (n = 20)
Laterality		
Left	11	12
Right	9	8
Localization		
Frontal	8	7
Temporal	5	4
Occipital	2	2
Fronto-temporal	1	2
Fronto-parietal	1	2
Parieto-occipital	2	2
Fronto-temporo-parietal	1	1
Maximal diameter of tumor		
>3.5 cm	9	11
<3.5 cm	11	9
Extension of resection		
Biopsy	4	5
Partial resection	12	11
Maximal resection	4	4
Supramaximal resection	0	0
Therapy		
Chemo-radiotherapy	4	6
Chemo-radiotherapy + TMZ	14	14
Chemo-radiotherapy+ TMZ + BEV	2	0

GBM-STS: glioblastoma short-term survivors, GBM-LTS: glioblastoma long-term survivors, TMZ: temozolomide, BEV: bevacizumab.

**Table 2:** Significance and ROC analysis of hsa-miR-424-5p, hsa-miR-216a-5p and hsa-miR-133a-3p based on normalized Ct values obtained from RT-qPCR, comparing GBM-LTS–GBM-STS, GBM-LTS–control and GBM-STS–control.

		hsa-miR-216a-5p	hsa-miR-133a-3p	hsa-miR-424-5p
GBM-LTS vs. GBM-STS	*p*-Value	0.00096	0.00064	0.0091
	AUC	0.81	0.82	0.74
	SE	0.75	0.95	0.45
	SP	0.8	0.6	1
GBM-LTS vs. Control	*p*-Value	0.0024	0.038	0.004
	AUC	0.78	0.69	0.77
	SE	0.7	0.65	0.9
	SP	1	0.7	0.7
GBM-STS vs. Control	*p*-Value	<0.00001	<0.00001	<0.00001
	AUC	1	0.96	0.89
	SE	1	1	1
	SP	1	0.85	0.7

**Table 3:** Information about the gender, age, and IDH status of the members of NGS cohort and (**a**) the patients included in the RT-qPCR validation group (**b**).

(**a**) **NGS Cohort**			
**Members of NGS**	**Gender**	**Age**	**IDH Status**
GBM-STS_1	M	55	IDH wild type
GBM-STS_2	M	50	IDH wild type
GBM-STS_3	M	67	IDH wild type
GBM-STS_4	F	59	IDH wild type
GBM-STS_5	F	64	IDH wild type
GBM-LTS_1	M	61	IDH wild type
GBM-LTS_2	M	59	IDH wild type
GBM-LTS_3	M	50	IDH wild type
GBM-LTS_4	M	63	IDH wild type
GBM-LTS_5	F	67	IDH wild type
GBM-LTS_6	F	64	IDH wild type
GBM-LTS_7	F	65	IDH wild type
GBM-LTS_8	F	64	IDH wild type
Control_1	M	72	-
Control_2	M	52	-
Control_3	F	64	-
Control_4	F	68	-
(**b**) **Validation Cohort**			
**Characteristic**	**GBM-STS**	**GBM-LTS**	**Control**
Number	20	20	20
Sex (M/F)	10/10	12/8	10/10
Age—Median (range)	59 (33–81)	57 (37–75)	66 (40–76)
IDH status	IDH wild type	IDH wild type	-

## Data Availability

The miRNA−seq data generated in this study are publicly available in the NCBI Gene Expression Omnibus (GEO) database under accession number GSE335138. All other data generated or analyzed during this study are included in this article and its Supplementary Materials.

## Supplementary Materials

The following supporting information can be downloaded at https://www.mdpi.com/article/10.3390/ncrna12050041/s1: Table S1: Twenty-one miRNAs identified by miRNA next-generation sequencing (NGS) were found to be significantly deregulated in GBM-STS tissue samples compared with the control group. The miRNAs are listed according to their expression levels; among them, 11 were downregulated and 10 were upregulated. Table S2: Eighteen miRNAs revealed by miRNA NGS analysis exhibited significant deregulation in GBM-LTS tissue samples relative to the control group. The miRNAs are ranked according to their expression levels, with 12 showing downregulation and 6 showing upregulation. Table S3: Sixteen miRNAs detected through miRNA NGS profiling were significantly deregulated in GBM-STS tissue samples compared with the GBM-LTS group. The miRNAs are listed according to their expression levels; among them, 4 were downregulated and 12 were upregulated.

## Abbreviations

AGO2	Argonaute RISC Catalytic Component 2
AKT1	AKT Serine/Threonine Kinase 1
ANOVA	Analysis of variance
AQP4	Aquaporin 4
AUC	Area under the curve
BAM	Binary alignment map
BFAR	Bifunctional apoptosis regulator
CI	Confidence interval
Ct	Cycle threshold
DE	Differentially expressed
DNA	Deoxyribonucleic acid
FASTQ	FASTQ sequence format
FDR	False discovery rate
FFPE	Formalin-fixed paraffin-embedded
GBM	Glioblastoma
GBM-LTS	Glioblastoma long-term survivors
GBM-STS	Glioblastoma short-term survivors
GO	Gene Ontology
GSC	Glioma stem cell
HIF-1α	Hypoxia-inducible factor 1 alpha
HR	Hazard ratio
HSD	Honestly significant difference
IDH1/2	Isocitrate dehydrogenase 1 and 2
KEGG	Kyoto Encyclopedia of Genes and Genomes
LGR5	Leucine-rich repeat-containing G protein-coupled receptor 5
MACF1	Microtubule Actin Crosslinking Factor 1
MAP1B	Microtubule-Associated Protein 1B
MGMT	O6-methylguanine-DNA methyltransferase
miRNA	MicroRNA
MMP	Matrix metalloproteinase
MT1-MMP	Membrane-type 1 matrix metalloproteinase
NF-κB	Nuclear factor kappa B
NGS	Next-generation sequencing
NSC	Neural stem cell
OS	Overall survival
PCA	Principal component analysis
PC1	Principal component 1
PC2	Principal component 2
PFS	Progression-free survival
PI3K	Phosphoinositide 3-kinase
PPI	Protein–protein interaction
PRC	Polycomb Repressive Complex
RECK	Reversion-Inducing Cysteine-Rich Protein With Kazal Motifs
RIN	RNA integrity number
ROC	Receiver operating characteristic
RT-qPCR	Reverse transcription quantitative polymerase chain reaction
SE	Sensitivity
SP	Specificity
SUMO2	Small Ubiquitin-Like Modifier 2
TCGA	The Cancer Genome Atlas
TERT	Telomerase reverse transcriptase
TGF-β	Transforming growth factor beta
TIMP3	Tissue inhibitor of metalloproteinases 3
TNRC6A	Trinucleotide repeat-containing gene 6A
TNRC6B	Trinucleotide repeat-containing gene 6B
WHO	World Health Organization
