# Supplementary material for: Identification of Survival-Associated miRNA Signatures in Glioblastoma Using Next-Generation Sequencing
Source: Noncoding RNA. 2026 Oct 19;12(5):41. doi: 10.3390/ncrna12050041 (PMC13708145; doi:10.3390/ncrna12050041)
Supplement: Supplementary file 1 [file ncrna-12-00041-s001.zip › ncrna-4558812-supplementary.pdf]

Supplementary Material

1 Supplementary Tables

Table S1. Twenty-one miRNAs identified by miRNA next-generation sequencing (NGS) were found to be significantly deregulated in GBM-STS tissue samples compared with the control group. The miRNAs are listed according to their expression levels; among them, 11 were downregulated and 10 were upregulated.

GBM-STS vs. Control			
**miRNA**	**Regulation**	**LogFC**	**p GBM-STS vs. Control**
**hsa-miR-331-5p**	down	-2.19	0.03
**hsa-miR-340-3p**	down	-2.70	0.002
**hsa-miR-495-3p**	down	-3.18	0.02
**hsa-miR-1296-5p**	down	-3.29	0.003
**hsa-miR-340-5p**	down	-3.64	0.002
**hsa-miR-9983-3p**	down	-3.70	0.01
**hsa-miR-128-3p**	down	-3.73	0.007
**hsa-miR-128-3p**	down	-3.79	0.009
**hsa-miR-219a-5p**	down	-3.86	0.03
**hsa-miR-330-5p**	down	-4.40	0.005
**hsa-miR-139-5p**	down	-5.08	0.0003
**hsa-miR-378e**	up	1.15	0.01
**hsa-miR-574-5p**	up	1.79	0.008
**hsa-miR-3913-5p**	up	1.89	0.02
**hsa-miR-501-5p**	up	2.71	0.03
**hsa-miR-4746-5p**	up	2.72	0.03
**hsa-miR-27a-5p**	up	2.73	0.01
**hsa-miR-146b-5p**	up	2.87	0.02
**hsa-miR-542-5p**	up	2.98	0.01
**hsa-miR-21-5p**	up	3.22	0.007
**hsa-miR-216a-5p**	up	3.24	0.02

Table S2. Eighteen miRNAs revealed by miRNA NGS analysis exhibited significant deregulation in GBM-LTS tissue samples relative to the control group. The miRNAs are ranked according to their expression levels, with 12 showing downregulation and 6 showing upregulation.

GBM-LTS vs. Control			
**Accession**	**Regulation**	**Log FC**	**p GBM-LTS vs. Control**
**hsa-miR-548k**	down	-1.85	0.03
**hsa-miR-340-5p**	down	-2.26	0.03

**hsa-miR-133a-3p**	down	-2.31	0.01
**hsa-miR-128-3p**	down	-2.63	0.04
**hsa-miR-9983-3p**	down	-2.65	0.04
**hsa-miR-221-5p**	down	-2.69	0.05
**hsa-miR-128-3p**	down	-2.72	0.04
**hsa-miR-412-5p**	down	-2.93	0.01
**hsa-miR-330-5p**	down	-2.98	0.03
**hsa-miR-365b-3p**	down	-3.08	0.01
**hsa-miR-139-5p**	down	-3.29	0.003
**hsa-miR-409-5p**	down	-4.85	0.003
**hsa-miR-151a-3p**	up	1.50	0.03
**hsa-miR-3661**	up	1.75	0.04
**hsa-miR-193a-3p**	up	2.69	0.01
**hsa-miR-1262**	up	2.79	0.03
**hsa-miR-589-5p**	up	2.93	0.003
**hsa-miR-6515-5p**	up	3.31	0.03

Table S3. Sixteen miRNAs detected through miRNA NGS profiling were significantly deregulated in GBM-STS tissue samples compared with the GBM-LTS group. The miRNAs are listed according to their expression levels; among them, 4 were downregulated and 12 were upregulated.

**GBM-STS vs. GBM-LTS**			
**Accession**	**Regulation**	**Log FC**	**p GBM-STS vs. GBM-LTS**
**hsa-miR-1296-5p**	down	-2.0508604	0.02547971
**hsa-miR-3681-5p**	down	-2.0789163	0.03237974
**hsa-miR-195-3p**	down	-2.1017575	0.038196966
**hsa-miR-495-3p**	down	-2.4465027	0.031647295
**has-miR-133a-3p**	down	-1.9481444	0.026247231
**hsa-miR-23b-3p**	up	1.0651197	0.030483179
**hsa-miR-378e**	up	1.2190946	0.0019300195
**hsa-miR-133b**	up	1.6997566	0.021155342
**hsa-miR-574-5p**	up	1.7845192	0.0025389725
**hsa-miR-580-3p**	up	1.8538187	0.008044148
**hsa-miR-181b-3p**	up	1.9840716	0.023785453
**hsa-miR-424-5p**	up	2.5212355	0.017985431
**hsa-miR-216a-5p**	up	2.5757918	0.025768543
**hsa-miR-424-3p**	up	2.799939	0.016377056
**hsa-miR-187-3p**	up	3.0035896	0.020821873
